# Effects of Acute Lateral Ankle Sprain on Spinal Reflex Excitability and Time-to-Boundary Postural Control in Single-Leg Stance

**DOI:** 10.3390/healthcare13020149

**Published:** 2025-01-14

**Authors:** Joosung Kim, Kyung-Min Kim

**Affiliations:** 1 Department of Health and Human Performance, Texas State University, San Marcos, TX 78666, USA; j_k145@txstate.edu; 2 Department of Kinesiology and Sport Sciences, University of Miami, Coral Gables, FL 33146, USA; 3 Department of Sport Science, Sungkyunkwan University, Suwon-si 16419, Republic of Korea

**Keywords:** fibularis longus, ankle sprain, single-leg balance, Hoffmann reflex, time-to-boundary

## Abstract

**Background/Objectives**: Acute lateral ankle sprain (ALAS) affects balance, often assessed by changes in traditional center of pressure (COP) parameters. Spatiotemporal measures of COP and time-to-boundary (TTB) analysis may offer improved sensitivity in detecting postural deviations associated with ALAS. However, the neurophysiological mechanism underlying these changes remains unknown. This study aimed to explore the effects of ALAS on spinal reflex excitability in the fibularis longus (FL) during single-leg balance and TTB parameters following ALAS. **Methods**: Fourteen participants with and without ALAS were recruited within 14 days from the onset of the injury. We assessed FL spinal reflex excitability and postural control during a single-leg stance. The primary outcomes included the H/M ratio, H-latency, and TTB parameters. For H-reflex testing, the peripheral electrical stimulation was delivered at the sciatic nerve before bifurcating into the tibial and common fibular nerve while participants maintained a single-leg balance position with the involved side of the limb. The TTB parameters of the medial–lateral (ML) and anterior–posterior (AP) directions of the mean, SD, and minimum were assessed, which indicate postural correction and strategies. **Results**: Patients with ALAS had a significantly lower AP-TTB minimum compared with healthy uninjured controls, with a moderate effect size (*p* = 0.039; *d* = −0.83). However, there was no significant difference in the H/M ratio (ALAS: 0.29 ± 0.16 vs. CON: 0.24 ± 0.10; *p* = 0.258) and H-reflex latency (ALAS: 34.6 ± 1.92 vs. CON: 33.8 ± 1.75 ms; *p* = 0.277); **Conclusions**: These results indicate that reflex control at the spinal level may have a minimal role in response to balance deficits following ALAS.

## 1. Introduction

Acute lateral ankle sprains (ALAS) commonly occur in the general and athletic population. Each year, approximately 3 million patients in the United States visit the emergency department for ankle sprain [1]. ALAS involves tears of the lateral ankle ligaments, particularly the anterior talofibular ligament and calcaneofibular ligament, accounting for 91.7% of the total injuries [1,2]. Ligament healing takes weeks to a year until it returns to the previous level of tensile strength [2]. Returning to regular physical activity without addressing residual symptoms after the injury may hinder successful treatment and rehabilitation. Even though ankle sprain is often considered an innocuous condition [3], the likelihood of reinjury is high, with a lingering problem of imbalance and muscle control impairments [4].

Impaired postural control, which can be defined as excessive postural sway during a balance task, such as a one-leg stance, appears before sustaining an injury and manifests as a risk factor for initial injury and recurrent sprain [5]. The center of pressure (COP) measures using a force plate, including mean velocity, area, and range, have shown balance impairments in individuals with ALAS [5,6]. While these temporal- or spatial-based outcomes are useful, these traditional measures may not be strong enough to detect postural impairment associated with ankle injuries [7]. Time-to-boundary (TTB) measures that integrate both temporal and positional aspects in COP analysis provide valuable data on postural control correction and strategies [7,8]. The TTB estimates the time it takes for COP excursions to reach and exceed the boundaries of the base of support (i.e., the area where the foot contacts the ground) if the current COP trajectory and velocity are maintained [7,8]. It is particularly sensitive under static balance conditions, as it assesses the ability to maintain balance within the constraints of the support base [7]. TTB has been used to detect impaired postural control in neurological [9,10] and musculoskeletal conditions [11], including ankle injuries [12,13]. The TTB may be more sensitive for detecting postural control deficits in ALAS during a simple balance task [14], offering a deeper understanding of the injury and its impact on postural control. However, research using TTB in ALAS is still limited.

Different factors contribute to effective postural control, including proprioceptive, muscular, tactile, visual, and vestibular systems [15]. While these factors are partly responsible for a proper balance, the interplay between the central nervous system (CNS) and joint-stabilizing muscles is critical in maintaining balance [16]. The muscle spindle provides constant feedback to activate and control balance, and the spinal motor neuron pool in the CNS serves as the final gate that determines muscle contraction during balance [15]. The role of the spinal reflex mechanism in postural control is critical after ankle injuries, and particularly, the fibularis longus (FL) muscle often compensates for the structural deficit (e.g., increased laxity) [17]. Yet, how the FL in the spinal pathway adapts to balance impairment following ALAS remains unknown, particularly during single-leg standing, despite the importance of understanding compensatory mechanisms in response to ALAS.

The H-reflex is a reproducible and reliable measure of reflexive activation in response to submaximal percutaneous electrical stimulation. The stimulation evokes both the afferent and the efferent α-motor action potentials [18,19]. The excitatory afferent signals in the spinal circuit ultimately result in a reflex output, known as the H-reflex amplitude, which is normalized to the M-wave (the compound activation of the efferent motor nerves evoked by the supramaximal electrical stimulation). The normalized H-reflex to the M-wave, known as the H/M ratio, indicates the spinal reflex excitability [20]. H-reflex latency, which assesses the conduction time (ms) from the stimulus artifact to the onset of the reflex response, provides information regarding the nerve conduction efficiency in the reflex arc [21]. However, previous research with ALAS has primarily conducted H-reflex testing in resting [22,23,24] or bipedal stances [25], which may not adequately account for the functional role of spinal reflex mechanisms in response to a balance challenge following an injury. Exploring H-reflex measures in a single-leg balance could offer new insights into neurophysiological adaptations to impaired postural control following ALAS, especially in the FL muscle, which is critical for ankle stabilization.

Therefore, the current study took an exploratory approach to investigate FL H-reflex measures (the H/M ratio and H-reflex latency) in conjunction with TTB postural control in individuals with ALAS during single-leg balance. Specifically, we aimed to (1) examine FL spinal reflex excitability during a single-leg balance in ALAS compared with healthy controls; (2) assess postural control impairment using TTB analysis; and (3) explore potential associations between the FL H/M ratio, TTB outcomes, and clinical symptoms, such as ankle swelling, pain, and functional deficit. The research questions for this study were whether spinal reflex excitability and TTB outcomes differ between individuals with ALAS and healthy controls and whether injury symptoms and impaired postural control are associated with changes in spinal reflex excitability.

## 2. Materials and Methods

The current study used a case–control design with groups with and without ALAS. The primary outcomes were spinal reflex excitability and static postural control. Spinal reflex excitability of the FL was assessed by H-reflex measures during single-leg balance. Static postural control was examined using a TTB balance during the same single-leg balance position. We evaluated acute injury symptoms of ankle pain, swelling, and functional loss as a secondary outcome.

### 2.1. Participants

Participants were recruited from the college community. We used the following criteria to determine the eligibility of participants in this study. The inclusion criteria for the ALAS group were as follows: (1) a recent history of lateral ankle sprains within two weeks, (2) the current functional deficit of the ankle, quantified by scores of 90% or less in foot and ankle ability measure (FAAM)–activity of daily living (ADL), and of 80% or less in FAAM-sports, and (3) no current lower-extremity injury other than a recent ankle sprain. The inclusion criteria for the control group were as follows: (1) no history of ankle sprain, (2) no episodes of ankle joint giving way, (3) no functional limitation of the ankle, determined by scores of 95% or greater in FAAM-ADL, and of 90% or greater in FAAM-sports, (4) no current lower-extremity injury, and (5) no history of lower-extremity injuries in the previous six months.

Participants in both groups were excluded if they had (1) any history of musculoskeletal injuries to the lower-extremity joints other than ankle sprain in the previous six months for the ALAS group, resulting in at least one interrupted day of physical activity, (2) any history of surgery in the lower extremity, (3) any history of lower back pain in the previous six months, (4) any history of a diagnosed neurological disorder, (5) hypersensitivity to electrical stimulation, and (6) inability to balance on the injured foot for 10 s. This study was approved by the author’s Institutional Review Board prior to enrollment, and all participants provided written informed consent before any research activity in this study.

### 2.2. Acute Injury Symptoms

The licensed athletic trainer implemented a standardized evaluation for acute injury symptoms [25]. Current ankle pain intensity was assessed using a visual analog scale (VAS) instrument, which is a reliable method to quantify the pain for ankle pain [26]. Participants marked their perceived pain level on a 10 cm horizontal line in length that indicated the intensity of pain from 0, “no pain”, to 10, “the worst imaginable pain.” Ankle swelling was assessed using the “figure of eight” method that measures a circumference in cm, the distance by tape encircling the subtalar and talar joint [27]. For self-reported functional assessment, foot and ankle ability measures (FAAM) were utilized to evaluate the level of functional deficit during their usual activities of daily living (ADL) and their sports-related activities from 0 to 100, with a lower percentage score indicating more functional loss [28]. The FAAM is a reliable, valid, and responsive self-reported outcome measure for assessing physical functions related to leg, foot, and ankle disorders. It demonstrates high internal consistency (α = 0.96 for the ADL and 0.98 for the sports subscale), strong reliability (ICC: 0.89 and 0.87, respectively), and responsiveness, with minimum clinically important differences of 8 and 9 points, respectively [28].

### 2.3. Spinal Reflex Excitability

**Figure 1:**
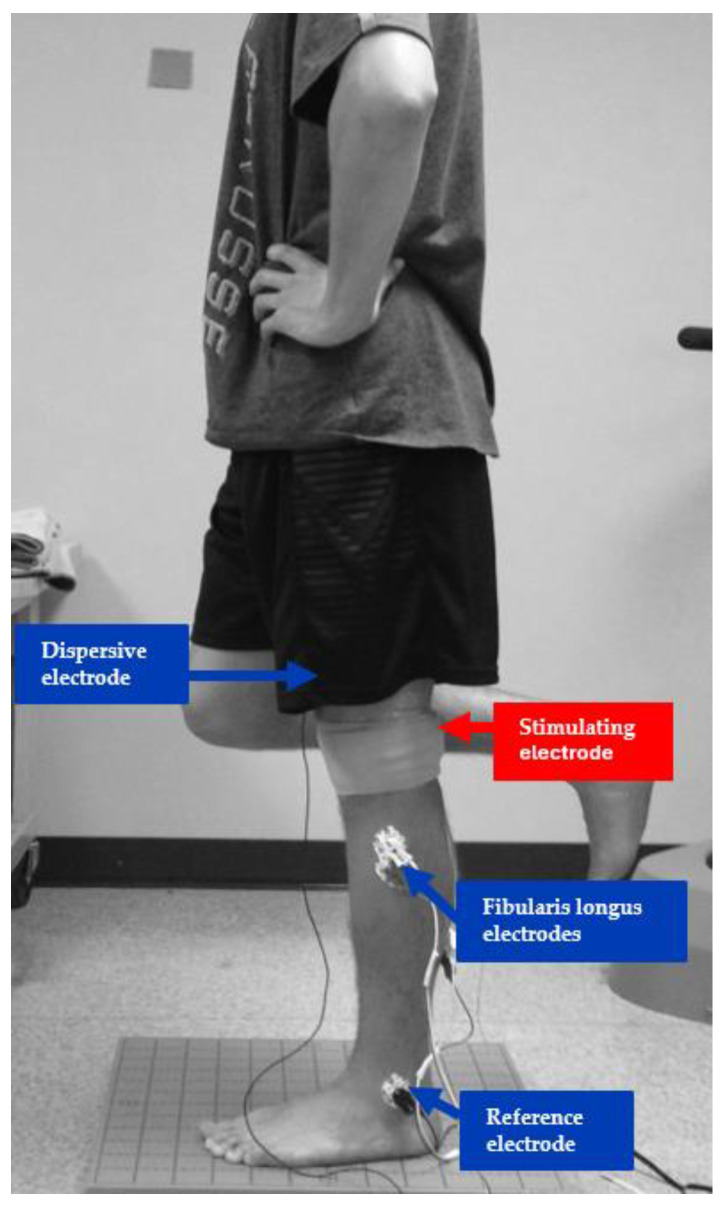
A set-up of Hoffmann reflex (H-reflex) testing during a single-leg stance.

The participants refrained from any anti-inflammatory medications, caffeine, alcohol, or tobacco 24 h before the testing session to prevent any potential influence on spinal reflex excitability [29]. Participants were lying prone on the table for the electromyography (EMG) setup before H-reflex testing. The skin over the FL area was shaved with a razor and abraded with abrasive skin preparation gel. Bipolar EMG electrodes with an Ag-AgCl contact surface with an 11 mm diameter and an inter-electrode space of 20 mm were placed over the FL and one reference electrode on the lateral malleolus according to the SENIAM recommendations [30]. The electrode impedance over the electrode placement area was controlled with an impedance level of <5 kΩ. EMG activity was further evaluated by performing eversion and plantarflexion of the foot to confirm the appropriate activity of the muscle. EMG signals were amplified at a gain of 1000, band-pass-filtered from 10 Hz to 500 Hz, and sampled at 2 kHz. The analog-to-digital signal was converted using a 16-bit converter (BIOPAC Systems, Goleta, CA, USA), and EMG signals were recorded using the Acknowledge software (Ver 5; BIOPAC Systems). For the stimulating electrodes, we secured the cathode (2 mm shield disk electrode, BIOPAC Systems Inc., Goleta, CA, USA) over the superior popliteal fossa proximal to the sciatic nerve before the bifurcation of the tibial and common peroneal nerve with a strip of hypoallergenic tape (3M Micropore, Maplewood, MN, USA) and placed another dispersive electrode (7 cm circular carbon-impregnated dispersive pad) over the anterior thigh just above the patella. This method is reliable with fibular longus H-reflex assessment [31] (shown in Figure 1). The H-reflex was performed while participants stood on one foot, maintaining a single-leg balance. The FL H-reflex was elicited by a 1 ms square-wave electrical stimulus applied to the sciatic nerve at increasing intensities until a maximal Hoffmann reflex (Hmax) and maximal motor response (Mmax) were found. The interstimulus interval was maintained at >0.1 Hz. The initial reference standing position was bipedal, transitioning to single-leg balance before stimulation upon the verbal cue from the investigator, maintaining it until the stimulation was felt, and gradually returning to the baseline position. A resting time of less than 3–5 min was provided if necessary. Five trials of Hmax and Mmax were recorded, and the average Hmax was normalized to the average Mmax to calculate the H/M ratio, representing spinal reflex excitability. H-reflex latency, defined as the time duration from the beginning of electrical stimulation to the onset of H-reflex, was analyzed in order to examine the amount of time it takes for the H-reflex to travel up to spinal motor neurons and then down to reach the muscle [20]. The H-reflex latency was normalized to leg length [32,33].

### 2.4. Static Postural Control

The static postural control was assessed using a force plate during the single-leg stance with eyes open on a force plate (AccuSway Plus, AMTI, Waterfront, MA, USA), in the same position as the spinal reflex excitability testing. We recorded three trials of a 10 s balance at a sample rate of 50 Hz [14]. The trial was discarded and repeated if a participant failed to maintain the standing posture with the opposite limb touching the floor, leaned against the stance limb, repositioned, or adjusted foot position. Center of pressure (COP) data were extracted and filtered with a fourth-order zero lag, low-pass Butterworth filter with a cutoff frequency of 5 Hz used for TTB analysis. A custom-made software program (Ver R2024a; MATLAB; The MathWorks Inc., Natick, MA, USA) was used to compute the TTB outcomes, such as means, standard deviations, and the minimum of the TTB minima in the mediolateral and the anteroposterior directions in the same manner as previously described in [8]. The base of support was drawn by measuring four distances in relation to the edge of the force plate surface, such as the 1st or 2nd distal phalanx, the distal portion of the calcaneus, the left-side head of the 5th, and the right side of the distance base of the support was measured from the left-side head of the 1st metatarsal. A smaller value of TTB parameters reflected a shorter time to correct postural control for the mean TTB, poorer postural adaptability for the TTB standard deviations, and the most extreme point of postural instability for the TTB minimum [7,8].

### 2.5. Statistical Analysis

The normal distribution of the data was assessed using Shapiro–Wilk tests, which found that two variables of postural control, such as the TTB-AP mean and TTB-ML SD, were non-normally distributed. Therefore, we used a mixed approach that selectively conducted non-parametric statistics for non-normally distributed outcomes. For the group comparison between ALAS and CON, separate independent *t*-tests were performed for normally distributed outcomes and the Mann–Whitney U test for non-normally distributed outcomes. Standardized Cohen’s *d*-effect sizes with 95% confidence intervals were calculated using pooled standard deviations to determine the magnitude of the mean difference between the groups. The strength of magnitude of Cohen’s *d* was interpreted as small (0.2), moderate (0.5), or large (*d* ≥ 0.8) [34]. For the relationship between the H/M ratio and latency and TTB, Pearson correlation (*r*) analysis was conducted for normally distributed outcomes, and the Spearman rho correlation (*rho-r_s_*) test was performed for non-normally distributed outcomes.

## 3. Results

### 3.1. Participant Characteristics

The descriptive data are presented in Table 1. Healthy individuals were well-matched to the ALAS patients for sex, age, height, and body mass. ALAS patients exhibited significant ankle swelling, pain (low level), and functional loss.

### 3.2. Spinal Reflex Excitability and Latency

The outcome measures for spinal reflex excitability are shown in Table 2. There was no significant difference in the pre-stimulus background EMG activity of the FL, indicating that the outcomes of the H/M ratio and H-reflex latency were not influenced by the level of muscle activity during standing (*p* = 0.319). The independent *t*-test revealed that there was no statistically significant difference in the H/M ratio (*p* = 0.258; *d* = 0.37) and H-latency (*p* = 0.999; *d* = 0.00) between the ALAS group and the control group during a single-leg balance, as shown in Table 2.

### 3.3. Postural Control

**Figure 2:**
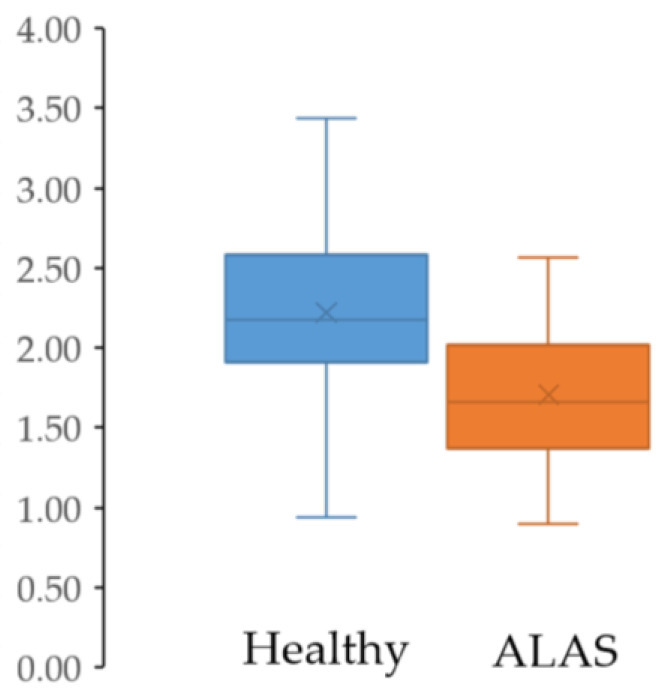
Box plot showing a significant difference in TTB-AP minimum between the healthy control and ALAS groups. The “×” represents the mean, while the midline within each box indicates the median.

For postural control, the independent *t*-test revealed that there was a significantly lower TTB-AP minimum in the ALAS group compared with CON with a large effect size (*p* = 0.039; *d* = −0.83) (Figure 2). Other TTB data were not significantly different between the groups (*p* > 0.05), as shown in Table 3.

### 3.4. Correlation Analyses

Correlational analyses revealed that there was no significant relationship between the FL H/M ratio and acute symptoms, self-reported outcomes, and postural control (*r* range: 0.048–0.324; *p* > 0.05), as shown in Table 4.

## 4. Discussion

This is the first study to comprehensively examine FL spinal reflex excitability in conjunction with TTB postural control during single-leg balance in individuals with ALAS. By examining these measures together, we aimed to gain more insight into neurophysiological and postural adaptations following ALAS, which may help develop targeted rehabilitation approaches. Our findings show that while patients with ALAS demonstrated diminished postural control, with a significantly lower TTB-AP minimum compared with healthy controls indicating a quicker postural boundary reach and instability, there was no noticeable difference in the FL H/M ratio and H-reflex latency between ALAS patients and healthy controls during a single-leg stance. Furthermore, no significant association was observed between these outcomes.

### 4.1. FL Spinal Reflex Excitability

The lack of a significant difference in the FL spinal reflex excitability aligns with previous research on resting position [22,23,24] and bipedal stance [25] following ALAS but contrasts with findings from an effusion study [35] and studies in individuals with CAI [36]. Hall et al. were the first to investigate spinal reflex excitability in ALAS patients, reporting that the FL H-reflex amplitude was not significantly different between injured and uninjured ankles [22]. In their study, they also found no changes in H-reflex latencies, and further correlation analysis revealed no significant association between ankle swelling and FL H-reflex latency [22]. This study used a within-study design, comparing only injured and uninjured limbs within the ALAS group. Later case–control research with healthy controls similarly demonstrated no side-to-side difference in the FL H/M ratio, as well as no difference between ALAS patients and controls [23,24]. A recent study examining postural control in a bipedal stance also found no alteration in the FL H/M ratio [25].

In contrast, an ankle joint effusion study reported an increase in FL H-reflex and M-responses, suggesting increased spinal excitability immediately after injecting saline into the joint capsule, in which a response lasted for up to an hour post-effusion [35]. The authors postulated that this facilitation might occur because of ankle muscle co-contraction, which stabilizes the joint position and prevents additional joint stress [35]. However, another study on ankle effusion utilizing EMG during functional stepping motion found no facilitation but inhibited neuromuscular activation in the presence of joint effusion [37]. These differences between actual injury and effusion studies may relate to the extent of edema, which diffuses into the interstitial space in actual ALAS injuries [35]. In individuals with CAI, a recent meta-analysis of seven studies involving 118 CAI and 117 uninjured controls found an overall reduction in the FL H/M ratio compared with controls [36]. However, the effect size was small (Cohen’s *d* = −0.27), and a study on both bipedal and unipedal stances did not show altered FL spinal reflex excitability between CAI and controls [38]. It should be noted that the soleus muscle has consistently presented an altered H/M ratio [23,24,25,36] in both ALAS and CAI. These differences between the FL and soleus may be attributed to their distinct roles in controlling posture and balance, as well as their morphological and physiological variations [23,39]. Collectively, these findings suggest that the FL spinal reflex excitability may not undergo early neuromuscular adaptation, unlike the soleus, and may play a minimal role in static balance.

### 4.2. Selective Postural Control Impairment After ALAS

Our TTB analysis further highlighted impaired postural control, specifically a reduced TTB-AP minimum, which reflects a quicker loss of postural stability in individuals with ALAS. Interestingly, other TTB parameters showed no significant differences. These findings may indicate a selective postural control deficit in response to ALAS. The absence of changes in the frontal-plane TTB measures, including mean, SD, and minimum values, may indicate rigid postural control strategies, likely due to ankle pain or a tendency to avoid frontal plane motion and mitigate reinjury risk [40]. Moreover, despite no statistical significance difference, there was a moderate effect size (*d* = 0.55) in TTB-AP AD. These findings differ from a previous study that observed altered TTB measures in the medial–lateral plane for the mean and SD within 24–72 h post-ALAS [14]. This discrepancy may arise from differences in post-injury timeframe (days). For instance, our study had an average post-injury period of 7.7 days, while the previous study [14] excluded individuals beyond 72 h post-injury. Nevertheless, our ALAS patients presented acute symptoms equivalent to the prior study [14]. This reduction in postural control impairment over the recovery period after ALAS has been observed in a longitudinal study [5] but needs to be tested with TTB measures throughout ALAS recovery.

### 4.3. Clinical Implications

ALAS frequently leads to balance impairments, affecting both static and dynamic postural control, with the risk of progressing to chronic instability if not properly addressed [2]. Interestingly, our findings indicate no significant difference in FL spinal reflex excitability between ALAS patients and healthy controls during single-leg stance. This suggests that spinal reflex adaptations in the FL may not play a critical role in the acute stage of balance recovery for ALAS, directing clinicians to focus on functional aspects of postural control, such as targeted proprioceptive and motor control training, rather than interventions aimed solely at modulating spinal reflexes [33].

Spinal reflex responses may evolve over time, and some individuals may develop chronic conditions such as CAI, as the body compensates for the ligament injuries and biomechanical changes. Therefore, our findings should be interpreted cautiously, and future research could benefit from measuring outcomes at multiple time points.

While balance training is widely recognized as an effective rehabilitation approach for patients with CAI, it has been less commonly implemented for those with ALAS. Current therapeutic strategies for ALAS patients generally focus on reducing acute symptoms through conventional methods, such as NSAIDs, taping, and icing [37]. Anecdotal evidence indicates that public awareness of the advantages of early balance training is limited, with many individuals opting to avoid weight-bearing on the injured ankle, preferring immobilization until symptoms are completely resolved. However, as much as the patient can tolerate, early mobilization may enhance recovery speed and restore ankle function, which was associated with increases in physical activity [38]. Our findings show that balance deficits can continue for one to two weeks following injury. The deficit seems initially present as multi-directional impairments [9] but narrows to more specific sagittal plane deficits as the injury heals. This highlights the potential benefits of a targeted, progressive exercise regimen aimed at addressing balance impairments.

### 4.4. Limitation

This study had several limitations. We compared the FL spinal reflex excitability and TTB postural control in individuals with ALAS with those in healthy controls, without examining these measures in the uninjured ankles of participants. Assessing the uninjured ankle could provide additional insight into the symmetry of neural and postural control measures. However, based on the previous observations [23,24,25], asymmetry in FL spinal reflex excitability is unlikely. This study did not record participants’ history of previous balance training; therefore, it is possible that some participants may have participated in such training previously. While the significant difference in postural control, as demonstrated by the AP TTB minimum, between individuals with ALAS and healthy controls suggests minimal potential influence from prior training history, neurophysiological data like the H/M ratio and H-reflex latency require more robustly controlled study conditions. Future studies should monitor participants’ training status to further minimize potential confounding factors. In addition, the current study involved a relatively small sample size. Studies on ALAS in this area have typically involved small sample sizes and homogeneous populations (young adults) due to logistic challenges in recruiting participants with ALAS. Future research could benefit from multicenter collaborations to improve sample diversity and size while overcoming logistic challenges in successfully recruiting larger ALAS samples. This approach would improve the statistical power and generalizability of the results and allow a more accurate interpretation of results regarding spinal reflex excitability and postural control parameters following ALAS, which ultimately guides an effective and targeted prescription of therapeutic interventions. Lastly, this study focused on static balance with a retrospective case–control design. For future studies, we recommend investigating other balance tasks, such as dynamic balance, using a prospective longitudinal study design.

## 5. Conclusions

The spinal reflex excitability of the fibularis longus was not altered after ALAS during single-leg balance, while impaired postural control was observed. These results indicate that reflex control at the spinal level of the fibularis longus may have a minimal role in response to balance deficits following ALAS.

## Figures and Tables

**Table 1:** Participant demographics (mean ± standard deviation).

Variables	ALAS (n = 14)	Healthy (n = 14)
Sex	5 males; 9 females	5 males; 9 females
Age (years)	19.8 ± 2.0	20.7 ± 2.3
Height (cm)	171.9 ± 8.2	174.2 ± 8.8
Mass (kg)	69.7 ± 8.2	69.5 ± 14.9
Time since injury (day)	6.3 ± 3.4	N/A
Swelling (cm)	1.1 ± 0.8	0.1 ± 0.2
VAS pain (cm)	2.6 ± 1.3	0.0
FAAM-ADL (%)	63.1 ± 11.5	99.9 ± 0.3
FAAM-sports (%)	38.3 ± 14.2	100.0 ± 0.0

Abbreviation: ALAS, acute lateral ankle sprain; N/A, not applicable.

**Table 2:** Spinal reflex excitability and latency of fibularis longus during single-leg standing (mean ± standard deviation).

Variable	ALAS	Healthy	Group Difference	Effect Size ^a^
H/M ratio	0.29 ± 0.16	0.24 ± 0.10	*t*(26) = 1.16; *p* = 0.258	*d* = 0.37 (−0.38, 1.11)
H-latency (ms)	37.9 ± 2.04	37.9 ± 1.81	*t*(26) = 0.00; *p* = 0.999	*d* = 0.00 (−0.74, 0.74)

Abbreviation: ALAS, acute lateral ankle sprain; ^a^ Cohen’s *d* estimate of effect size was calculated between two groups using pooled standard deviation, along with its associated 95% confidence interval.

**Table 3:** Postural control during single-leg standing (mean ± standard deviation).

Variable	ALAS	Healthy	Group Difference	Effect Size ^b^
TTB-ML mean (s)	1.74 ± 0.51	1.73 ± 0.41	*t*(26) = 0.03; *p* = 0.974	*d* = 0.02 (−0.72, 0.76)
TTB-AP mean (s) ^a^	3.13 ± 0.95	3.34 ± 0.94	*U* = 77.5; *p* = 0.358	*d* = −0.22 (−0.96, 0.53)
TTB-ML SD (s) ^a^	1.68 ± 0.97	1.39 ± 0.82	*U* = 91.5; *p* = 0.777	*d* = 0.32 (−0.43, 1.06)
TTB-AP SD (s)	1.60 ± 0.89	1.20 ± 0.49	*t*(26) = 1.47; *p* = 0.154	*d* = 0.55 (−0.23, 1.30)
TTB-ML minimum (s)	0.60 ± 0.21	0.66 ± 0.16	*t*(26) = 0.80; *p* = 0.428	*d* = −0.32 (−1.07, 0.45)
TTB-AP minimum (s)	1.70 ± 0.48	2.22 ± 0.75	*t*(26) = 2.18; *p* = 0.039	*d* = −0.83 (−1.57, −0.03)

Abbreviation: ALAS, acute lateral ankle sprain; TTB, time-to-boundary; ML, medial–lateral; AP, anterior–posterior; SD, standard deviation. ^a^ indicates a non-normally distributed outcome measure, and Mann–Whitney U tests were performed. ^b^ Cohen’s *d* estimate of effect size was calculated between two groups using pooled standard deviation, along with its associated 95% confidence interval.

**Table 4:** Correlation analysis of spinal reflex excitability of fibularis longus with acute symptoms, functional ability, and postural control in patients with acute lateral ankle sprain.

	H/M Ratio	
	*r/rho-rs*	*p*
Acute symptoms		
VAS for pain (cm)	0.135	0.645
Ankle swelling (cm)	−0.206	0.480
Functional ability		
FAAM-ADL (%)	−0.159	0.588
FAAM-sport (%)	−0.185	0.527
Postural control		
TTB-ML mean (s)	0.203	0.486
TTB-AP mean (s) ^a^	−0.130	0.658
TTB-ML SD (s) ^a^	0.105	0.720
TTB-AP SD (s)	−0.325	0.257
TTB-ML minimum (s)	0.113	0.700
TTB-AP minimum (s)	0.048	0.872

^a^ indicates non-normally distributed outcome measure, and the Spearman rho correlation test (*rho-rs*) was performed.

## Data Availability

The data are available from the corresponding author upon request. The data are not publicly available due to privacy and ethical restrictions.
